# Efficacy and safety of Shenfu injection combined with sodium nitroprusside in the treatment of chronic heart failure in patients with coronary heart disease: A protocol of randomized controlled trial

**DOI:** 10.1097/MD.0000000000024414

**Published:** 2021-02-19

**Authors:** Binbin Guo, Tian Yang, Jinniang Nan, Qianghui Huang, Chenxiu Wang, Wenpeng Xu

**Affiliations:** a Department of Clinical Medicine, Jiangxi Health Vocational College of China; b Second Affiliated Hospital of Nanchang University; c Jiangxi Provincial People's Hospital; d Jiangxi Provincial Traditional Chinese Medical Hospital, Nanchang, Jiangxi Province, China.

**Keywords:** chronic heart failure, coronary heart disease, randomized controlled trial, Shenfu injection, sodium nitroprusside

## Abstract

**Background:**

Coronary heart disease is a serious cardiovascular disease. There is coronary atherosclerosis, resulting in lumen stenosis, blockage, and then the symptoms of insufficient blood supply and hypoxia in the myocardium. Chronic heart failure is a kind of syndrome with abnormal ventricular filling and ejection function, which is the final stage of the development of coronary heart disease. At present, the treatment plan of Western medicine can significantly reduce the hospitalization rate, but it is still not satisfactory for the prognosis and mortality of patients. Shenfu injection has advantages in the treatment of heart failure in patients with coronary heart disease, but there is a lack of standard clinical studies to verify it, so the purpose of this randomized controlled study is to evaluate the efficacy and safety of Shenfu injection combined with sodium nitroprusside in the treatment of chronic heart failure in patients with coronary heart disease.

**Methods:**

This is a prospective randomized controlled trial to study the efficacy and safety of Shenfu injection combined with sodium nitroprusside in the treatment of chronic heart failure in patients with coronary heart disease. The patients will be randomly divided into a treatment group and the control group according to 1:1, in which the treatment group is treated with Shenfu injection combined with sodium nitroprusside, and the control group is treated with sodium nitroprusside alone. Both groups will be treated with standard treatment for 7 days and followed up for 30 days to pay attention to their efficacy and safety indexes. The observation indexes include TCM syndrome score, N-terminal pro-brain natriuretic peptide, left ventricular ejection fraction, brain natriuretic peptide, left ventricular end-systolic diameter, left ventricular end-diastolic diameter, stroke volume, adverse reactions and so on. We will use SPSS 25.0 software for data analysis.

**Discussion:**

This study will evaluate the efficacy and safety of Shenfu injection combined with sodium nitroprusside in the treatment of chronic heart failure in patients with coronary heart disease. The results of this experiment will provide a clinical basis for Shenfu injection combined with sodium nitroprusside in the treatment of chronic heart failure in coronary heart disease.

**Trial registration:**

DOI 10.17605/OSF.IO/4KNG3

## 1 Introduction

Chronic heart failure is characterized by abnormal cardiac structure and function, which lead to the decrease of ventricular ejection fraction and ventricular filling, which cannot meet the metabolic needs of body tissue. It is a critical cardiac disease and the main cause of death in heart patients.^[1]^ The incidence of heart failure in China is 0.9%, which is higher in women than in men, higher in the north than in the south, and higher in cities than in rural areas.^[2]^ The European Society of Cardiology found that there are more than 15 million patients with heart failure for every 1 billion people, with a total prevalence rate of 2% to 3% in the general population and 10% to 20% in people aged 70 to 80.^[3]^

Sodium nitroprusside has a certain value in improving the prognosis of patients with chronic heart failure.^[4]^ Sodium nitroprusside is a vasoactive drug, which can effectively dilate small arteries and veins, selectively relax vascular smooth muscle and reduce peripheral vascular resistance, temporarily change hemodynamic variables, reduce blood pressure, relieve cardiac load, and increase cardiac output.^[5]^ However, long-term use of nitric oxide donor sodium nitroprusside can lead to outflow tissue damage through protein nitrification, resulting in an increase in intraocular pressure, and drug withdrawal cannot recover.^[6]^

Traditional Chinese medicine in the treatment of chronic heart failure in patients with coronary heart disease can effectively alleviate the symptoms of heart failure and improve the quality of life of patients.^[7,8]^ According to the theory of traditional Chinese medicine, the main pathogenesis of chronic heart failure is yang-deficiency of heart and kidney.^[9]^ Shenfu injection is made from ginseng and aconite extract. Modern medical research has found that Shenfu injection can reduce peripheral vascular resistance, improve microcirculation,^[10]^ protect cardiomyocytes,^[11]^ and protect vascular endothelium.^[12]^ At the same time, Shenfu injection has small clinical adverse reactions and a safe wide range of dose. Therefore, we intend to use this randomized controlled trial to evaluate the efficacy and safety of Shenfu injection combined with sodium nitroprusside in the treatment of chronic heart failure in patients with coronary heart disease.

## 2 Materials and methods

### 2.1 Study design

**Figure 1:**
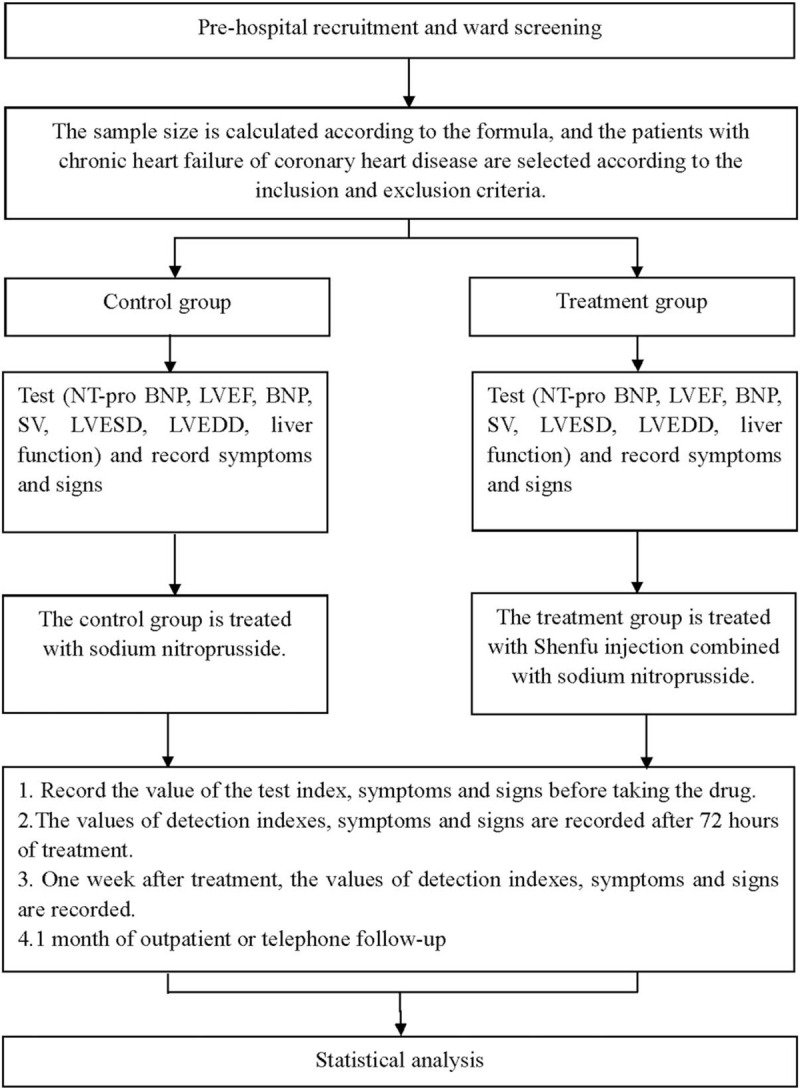
Flow diagram.

This is a prospective randomized controlled trial to study the efficacy and safety of Shenfu injection combined with sodium nitroprusside in the treatment of coronary heart disease with chronic heart failure. This trail will follow the comprehensive test reporting standards, and the flow chart is shown in Fig. 1.

### 2.2 Ethics and registration

This research scheme is in line with the Helsinki Declaration and approved by the Clinical Research Ethics Committee of our hospital. This research scheme has been registered on OSF (registration number: DOI 10.17605/OSF.IO/4KNG3). Before being randomly divided into groups, all patients need to sign a written informed consent form, and they are free to choose whether to continue the trial at any time.

### 2.3 Patients

Inclusion criteria: meet the diagnostic criteria of coronary heart disease, refer to the 2017 Chinese guidelines for Prevention and treatment of Cardiovascular Diseases; meet the diagnostic criteria of chronic heart failure,^[13]^ Doppler ultrasound indicates that the left ventricular ejection fraction is less than 50%,^[14]^ and the condition was in stable stage; age 40 to 79 years old; all patients signed consent.

Exclusion criteria: patients with other serious cardiovascular diseases; patients with other serious diseases such as malignant tumors; patients with severe mental illness; patients who are allergic to or have contraindications to medication in this study; unable to understand the study plan or unwilling to participate after explanation.

### 2.4 Sample size calculation

This study was a pilot clinical trial. According to the analysis and calculation of the related literature published in the past 10 years, the therapeutic efficiency of Western medicine is 72.3%, and the effective rate of traditional Chinese medicine combined with Western medicine is 92.1%. Using the sample size estimation formula compared with multiple sample rates, set the significance test level α = 0.05, then μ_α_ = 1.96, 1−β = 0.9, μ_β_ = 1.28. δ = π_1_−π_2_ sample size estimation formula for comparison of 2 sample rates:

$$N_{1}=N_{2}={\left(\frac{\mu_{\alpha }+\mu_{\beta }}{\delta }\right)}^{2}[\pi_{1}(1-\pi_{1})+\pi_{2}(1-\pi_{2})]$$

The sample size of each group is calculated to be 73. Considering shedding and other factors, the expansion is about 20%, and it is calculated that 182 patients need to be included. According to the simple randomized method, the patients included in the study are divided into control group (n = 91) and the observation group (n = 91).

### 2.5 Study design

In this study, through prehospital recruitment, in-hospital inpatients to screen patients who meet the criteria, patients and their families approve the study program and sign the informed consent form. The patients will be randomly assigned to the treatment group and the control group. Among them, the treatment group will receive Shenfu injection (Shenfu injection 40 mL injection + 5% GS 250 mL injection, ivgtt, QD) combined with intravenous drip of sodium nitroprusside (sodium nitroprusside 3 μ g / kg/min), while the control group will only receive intravenous drip of sodium nitroprusside. Patients in both groups will receive the same routine care and drug treatment, such as angiotensin converting enzyme inhibitor/angiotensin II receptor blocker, beta blocker, diuretic, etc. If necessary, the attending physician can adjust the program according to the patient's condition, and all interventions will be recorded in detail for final result analysis. We will set up a special drug manager, and the nurse will be responsible for preparing the medicine and preparing the solution. Therefore, the study is not blind to nurses, but to researchers, patients, and statisticians. All patients will receive an intravenous injection once a week. The health status of each patient is evaluated before and after treatment, including efficacy and safety indicators, and all patients are followed up by telephone for 30 days. Follow-up included cardiovascular events and rehospitalization.

### 2.6 Evaluation criteria and judgment of curative effect

1. Main outcome indicators: TCM syndrome score (refer to the guiding principles of Clinical Research of New drugs of traditional Chinese Medicine^[15]^), N-terminal pro-brain natriuretic peptide (NT-pro BNP), left ventricular ejection fraction, brain natriuretic peptide (BNP).
2. Secondary outcome indicators: stroke volume (SV), left ventricular end-systolic diameter, left ventricular end-diastolic diameter.
3. Adverse reactions: including abnormal liver function and uncomfortable symptoms (such as dizziness, nausea, etc) during treatment.

### 2.7 Data collection and management

The data are collected according to the evaluation criteria before the beginning of treatment, 72 hours after the treatment and at the end of treatment. Thirty days after the end of treatment, each patient is followed up by outpatient or telephone. The reasons for the loss of follow-up cannot be collected. All data will be collected jointly by 1 or 2 assistants. Personal information about potential participants and registered participants will be collected, shared, and stored in a separate storeroom to protect before, during, and after confidentiality. The access to the database is limited to the researchers of this research group.

### 2.8 Statistical analysis

In this study, SPSS25.0 statistical analysis software is used for data analysis, and the measurement data are expressed by ($\bar{\text{x}}\pm \text{S}$). For the data that accord with normality and homogeneity of variance, group *t* test is used between groups, paired *t* test is used within groups, rank sum test is used for those who do not conform to normal distribution, and *χ*^2^ test is used for counting data. When *P* *<* 0.05, it is statistically significant.

## 3 Discussion

Chronic heart failure is a common manifestation of coronary heart disease and an end-stage manifestation of all heart diseases.^[16]^ In the increasingly severe trend of population aging in China, the incidence rate is increasing. The proportion of patients with chronic heart failure caused by coronary heart disease is about 46.8%, with high morbidity and mortality.^[17]^ Its main manifestation is the decrease of ventricular filling and pumping function.^[18]^ At present, Western medicine is mainly treated with cardiotonic drugs, diuretics, and vasodilators, which can reduce the hospitalization rate and risk factors to a certain extent. But it still cannot achieve the ideal effect.^[19]^ In recent years, traditional Chinese medicine has accumulated rich experience in the treatment of chronic heart failure, and the effect is remarkable, which can significantly improve the symptoms of patients and delay the development of the disease.^[20]^ Most patients with heart disease have sleep disorders. A study shows that massage and aromatic massage can improve the sleep quality of these patients.^[21]^ The combination of traditional Chinese and Western medicine in the treatment of chronic heart failure of coronary heart disease has become a trend of clinical treatment.

Ginseng and aconite, the main components of Shenfu injection, have the effect of restoring yang and saving adversity, replenishing qi, and relieving detoxification, which has been significantly effective in the treatment of heart failure of coronary heart disease for a long time, and its protective effect on heart has been proved.^[22]^ Ginsenosides, the main active ingredient in ginseng, account for 4% of the total ginseng,^[23]^ which can improve the immunity of the body; the active ingredients of aconite have cardiotonic and antiarrhythmic effects;^[24]^ and the combination of the 2 drugs can protect blood vessels and heart.^[25]^ Myocardial ischemia-reperfusion in patients with coronary heart disease can cause aseptic inflammation. The participation of inflammatory mediators will lead to persistent injury of cardiomyocytes, and toll-like receptors are the receptors of inflammatory pattern recognition.^[26–29]^ Shenfu injection can significantly reduce the recognition of Toll-like receptor inflammation and the expression of inflammatory factors, reduce cardiomyocyte injury, improve cardiac function, and less adverse clinical reactions.^[30]^

The purpose of this randomized controlled trial is to verify that Shenfu injection can significantly improve the clinical symptoms and cardiac function in patients with chronic heart failure in patients with coronary heart disease. Since there is no standard clinical study to evaluate the efficacy of Shenfu injection combined with sodium nitroprusside in patients with heart failure of coronary heart disease, we intend to evaluate its efficacy and safety through prospective randomized controlled trials.

This study also has some limitations: due to the short planned follow-up time, we are unable to understand the impact of long-term results, so we may extend the follow-up time if necessary. And due to the influence of treatment, this study cannot achieve strict double-blindness, which may affect the results to a certain extent.

## Author contributions

**Data collection:** Binbin Guo and Tian Yang

**Funding acquisition:** Wenpeng Xu.

**Funding support:** Wenpeng Xu

**Investigation:** Tian Yang and Jinniang Nan

**Resources:** Jinniang Nan and Qianghui Huang

**Software operating:** Qianghui Huang

**Software:** Qianghui Huang.

**Supervision:** Chenxiu Wang and Wenpeng Xu

**Writing – original draft:** Binbin Guo and Tian Yang

**Writing – review & editing:** Binbin Guo and Wenpeng Xu
